# Molecular characterization and nutritional regulation of sodium-dependent glucose cotransporter 1 (Sglt1) in blunt snout bream (*Megalobrama amblycephala*)

**DOI:** 10.1038/s41598-021-93534-9

**Published:** 2021-07-07

**Authors:** Hualiang Liang, Xianping Ge, Mingchun Ren, Lu Zhang, Dong Xia, Ji Ke, Liangkun Pan

**Affiliations:** 1 Key Laboratory for Genetic Breeding of Aquatic Animals and Aquaculture Biology, Freshwater Fisheries Research Center (FFRC), Chinese Academy of Fishery Sciences (CAFS), Wuxi, 214081 China; 2 Wuxi Fisheries College, Nanjing Agricultural University, Wuxi, 214081 China; 3 Tongwei Co., Ltd., Chengdu, 610093 China; 4 Healthy Aquaculture Key Laboratory of Sichuan Province, Chengdu, 610093 China

**Keywords:** Molecular biology, Physiology

## Abstract

Fish has poor utilization capacity for glucose metabolism. The possible reasons are related to the core regulatory elements of glucose metabolism: transport proteins. Studies on the species and functions of Sglt1 in aquatic animals are scarce, therefore further studies are needed. In this study, the full length of blunt snout bream (*Megalobrama amblycephala*) *sglt1* (*Masglt1*) was 2965 bp including 5′-UTR region of 168 bp and a 3′-UTR region of 820 bp. *Masglt1* have a highest sequence homology in Cypriniformes fish. MaSglt1 protein was identified as a transmembrane protein with 14 α-helix structures locating plasma membrane by the methods of predicted tertiary structure and immunohistochemical staining. MaSglt1 protein has a hollow channel forms which could be specifically coupled with two Na^+^ ions to recognize glucose and carry out transmembrane transport. High *sglt1* mRNA was found in the intestine and kidney. The mRNA levels of intestinal *sglt1* had a positive correlation with dietary starch levels at 3 h after feeding, and the mRNA was significantly higher than that at 24 h, however, the mRNA levels of renal *sglt1* presented results opposite to those of intestinal *sglt1*. The mRNA levels of renal *sglt1* had a positive correlation with dietary starch levels at 24 h after feeding, and the expression was significantly higher than that at 3 h. These results confirmed that *Masglt1*1 was mainly found in the intestine and kidney and was located in the cell membrane, playing a role in glucose homeostasis.

## Introduction

In mammals and aquatic animals, glucose, playing a key role in energy supply, is a necessary metabolic substrate in physiological process. Mammals and aquatic animals are able to hydrolyze ingested disaccharides and polysaccharides obtained directly from the diet^1–3^. The intestine is an important organ for glucose metabolism in mammals and aquatic animals and plays a key role in metabolism processes involving the absorption and transport of glucose^4,5^. Several studies have reported that digestion of complex carbohydrates in mammals mainly depends on the physiological effect of pancreatic enzymes and brush border hydrolases of the intestine^6^. Similar processes in fish also occur, as complex polysaccharides are converted into monosaccharides, and a large number of carbohydrate monomers are absorbed^7^. In terms of the regulatory mechanism of absorption, glucose accumulates within the epithelium across the brush border membrane first, however, the process of the lumen of the small intestine to target cells depends on the role of transport systems^6,8^. However, the utilization of carbohydrates by fish varies^9^, and fish has poor utilization capacity for glucose metabolism. There were inability to digest high dietary carbohydrate, if the levels are too high, persistent hyperglycemia and growth decline would be observed in fish^10–13^. The possible reasons are related to the core regulatory elements of glucose metabolism: transport proteins^3^.

According to a previous study, specific systems of membrane transport play an extremely important role in the processes of monosaccharide products transported into enterocytes^14^. Previous study showed that sodium-dependent glucose cotransporter 1 (SGLT1), a component of the main intestinal membrane transport system, plays a key role in intestinal glucose absorption, and the regulation of this function has been studied in detail in mammals^15,16^. Hediger et al. (1987) cloned rabbit SGLT1 for the first time in 1987^17^. Subsequently, SGLT1 genes of humans^18^, insects^19^, rats^20^, sheep^21^ and other species have since been cloned. Like the mechanism in mammals, some fish species, such as gilthead sea bream (*Sparus aurata*)^22^ and common carp (*Cyprinus carpio*)^23^, have also been reported that *sglt1* plays an important role in the absorbed function from brush border membrane vesicles (BBMVs) isolated in the intestine. Furthermore, some studies have shown that *sglt1* is also expressed in the kidney of shark (*Squalus acanthias*)^24^ and skate (*Leucoraja erinacea*)^25^ and it has similar functional regulation as in the intestine of fish^22,23^, which is mainly used by the kidney to reabsorb glucose^26^. However, compared with mammalian studies, functions of Sglt1 in aquatic animals are scarce, and further studies are needed.

Blunt snout bream (*Megalobrama amblycephala*), an important economic fish in China, is very popular among Chinese consumers because of its good meat quality^27^. Like other fish, blunt snout bream has poor utilization capacity for glucose metabolism. If the dietary carbohydrate levels are too high, persistent hyperglycemia would be observed in blunt snout bream^13^. However, the relative mechanisms involved in carbohydrate intolerance are unclear, hence, we urgently need to carry out research in this aspect. The purpose of this experiment was to clone and identify functional characterization and nutritional regulation of Sglt1 in blunt snout bream.

## Results

### Biological analysis of blunt snout bream SGLT1 cDNA (MaSGLT1)

**Figure 1:**
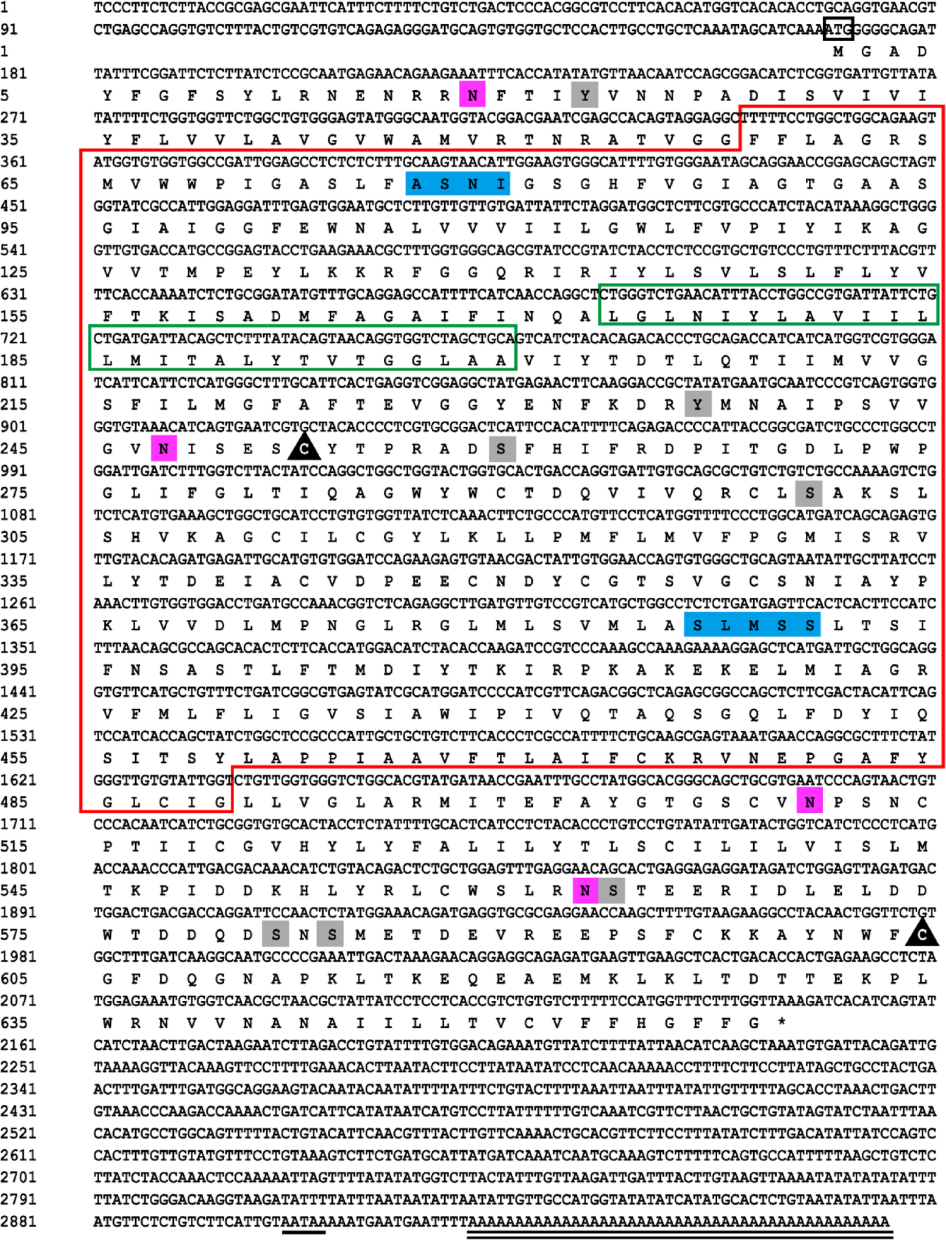
Sequencing results and amino acid sequence analysis of Sglt1 in *Megalobrama amblycephala*. The black box represents the ATG start codon; * represents the stop codon; underline represents the Poly(A) tail signal; double underline represents the poly(A) sequence; red box represents possible SSF domains (pfam00474); green box represents SSF protein characteristic conserved site NA_SOLUT_symp_1 (IPR018212); blue shadow represents Na^+^ specific coupling sites; purple shadow represents the possible N glycosylation sites; gray shadow represents possible phosphorylation sites; black triangles represents possible disulfide bond formation sites.

**Figure 2:**
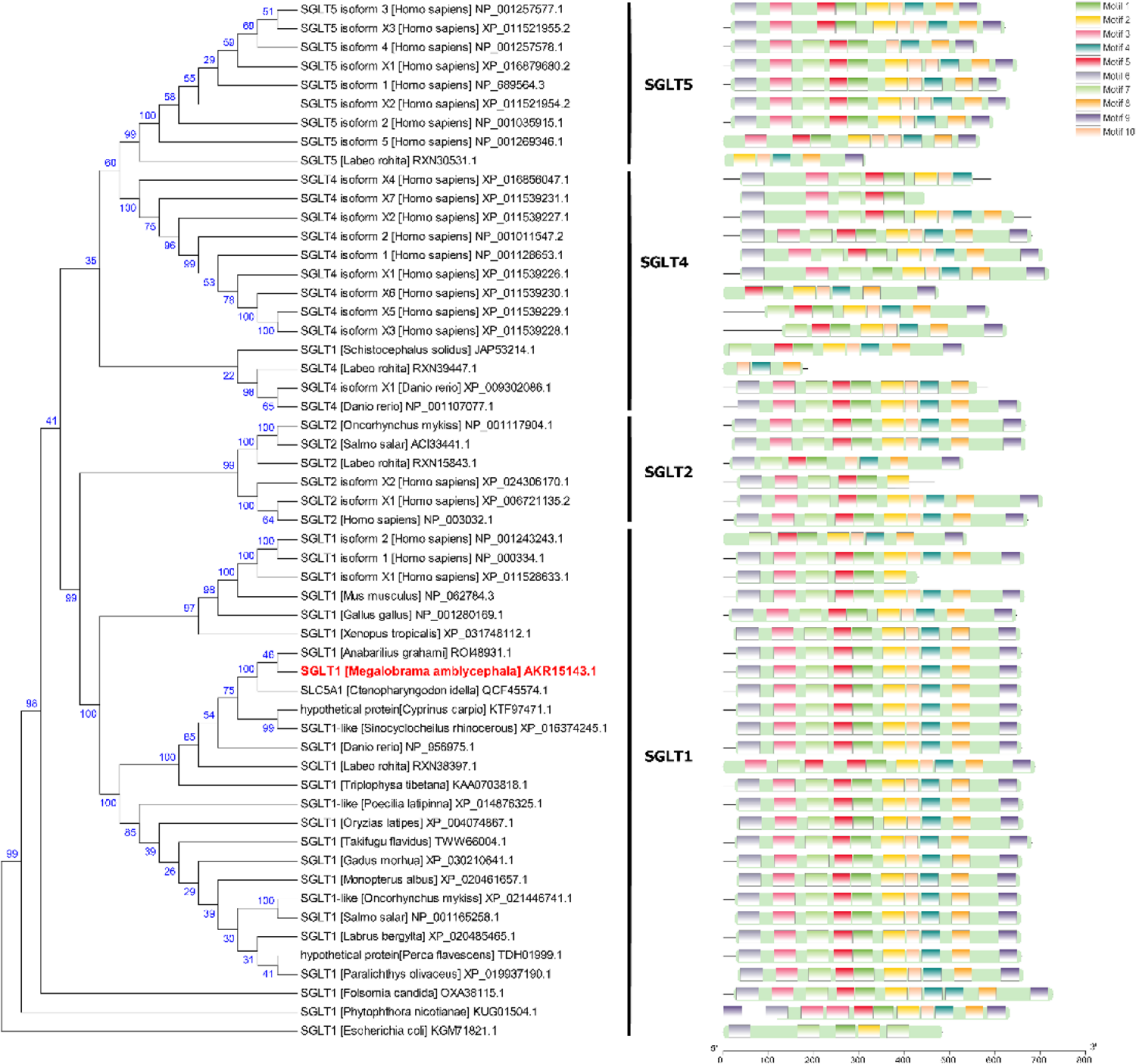
Evolutionary relationships of SGLT, domain and motif analyses of SGLT1 in *Megalobrama amblycephala* (GenBank accession no: AKR15143.1) and other species.

**Figure 3:**
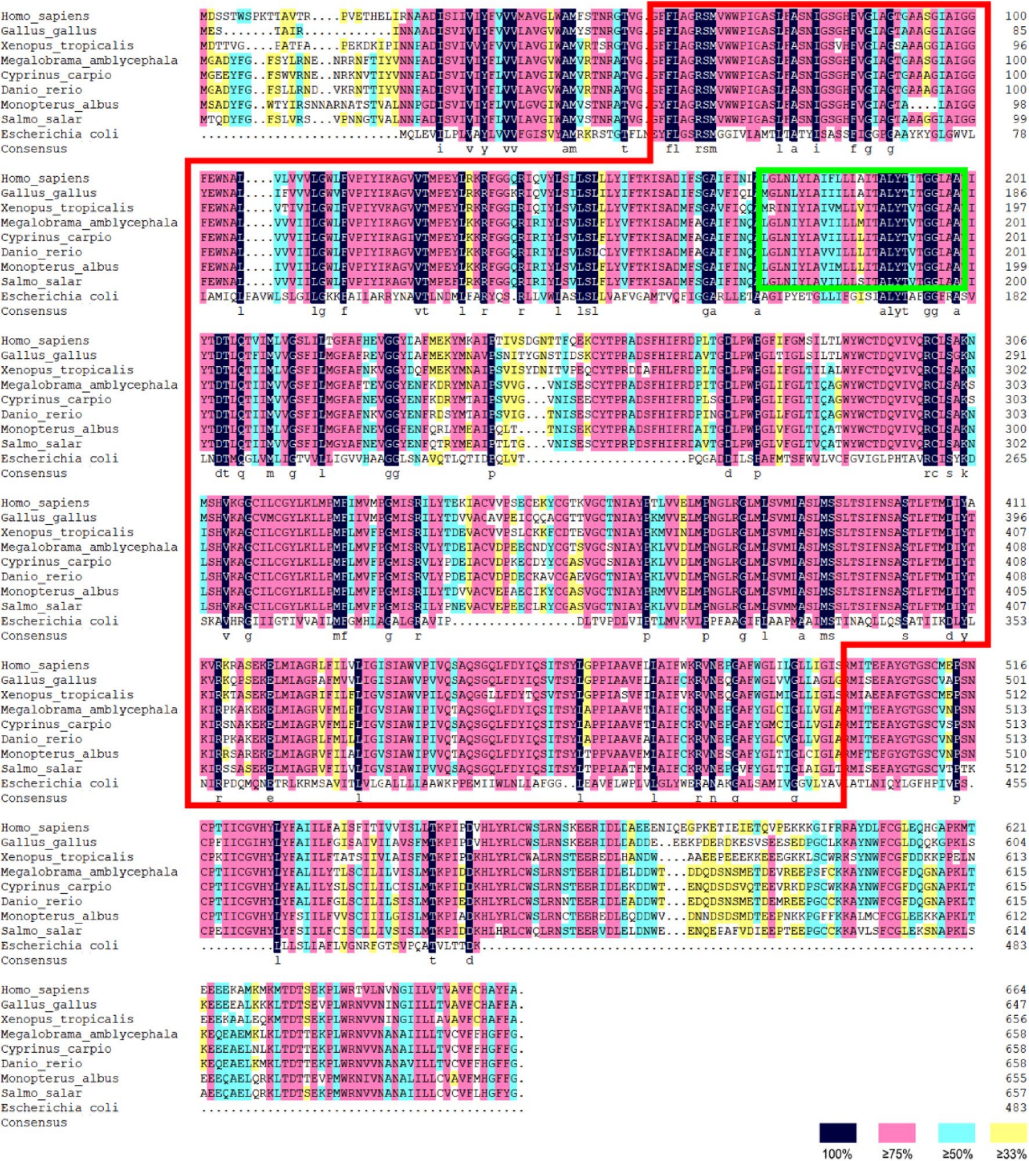
Homologous alignment of SGLT1 amino acid sequences in different species. The red box represents the SSF domain (PFAM00474), green box represents SSF protein characteristic conserved site NA_SOLUT_symp_1 (IPR018212).

**Figure 4:**
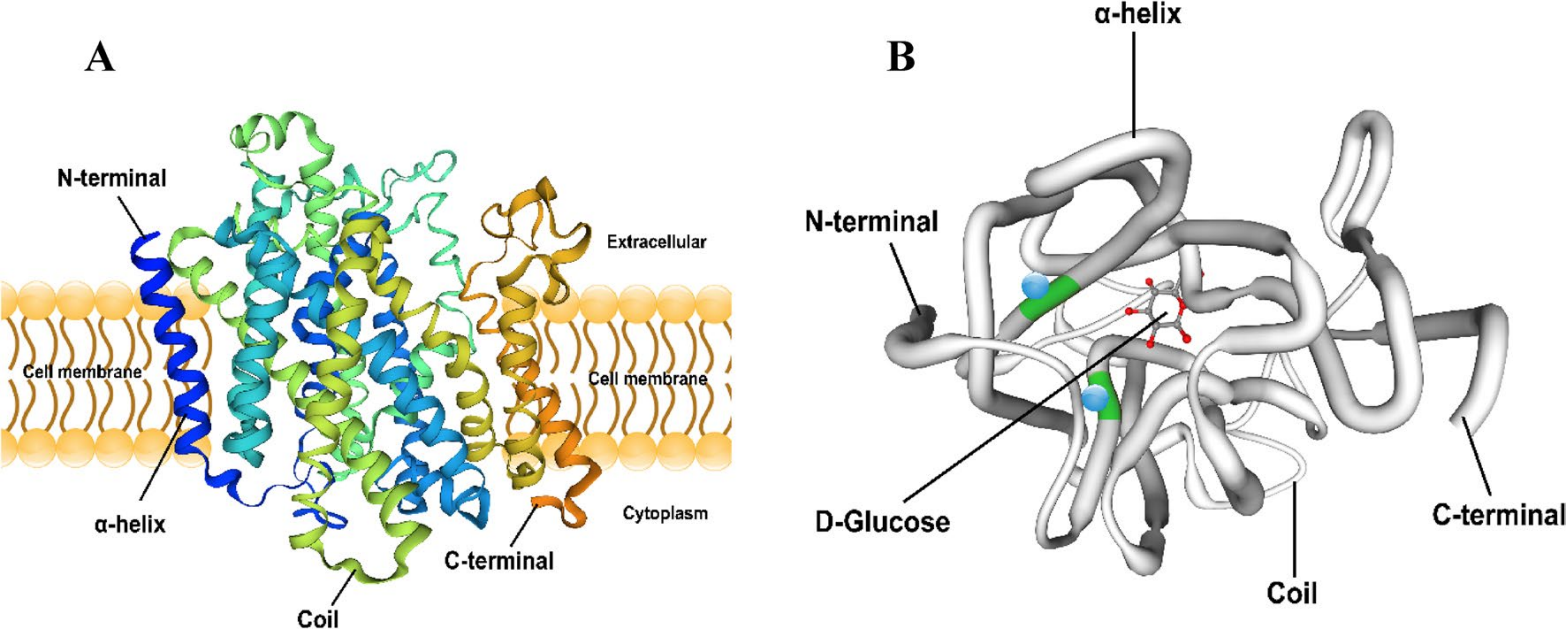
Protein 3D structure prediction of Sglt1 in *Megalobrama amblycephala.*

1. The full length of *Masglt1* was 2965 bp including 5′-UTR region of 168 bp and a 3′-UTR region of 820 bp. Functional analysis of the *sglt1* cDNA sequence was performed, and localization of the obvious AATAA site (polyA tail signal) and poly(A) structure were determined (Fig. 1). The *Masglt1* cDNA sequence has an open reading frame of 1977 bp, encoding 658 amino acids (Fig. 1). The 6.14 and 72.97 kDa were theoretical isoelectric point and molecular weight of *Masglt1*, respectively. The amino acid sequence of the molecular formula of Sglt1 is C_3363_H_5200_N_818_O_908_S_42_, with 50 basic amino acids (Asp + Glu) and 47 acidic amino acids (Arg + Lys); the extinction coefficient is 115,835, and the absorption coefficient is 1.588. The instability coefficient is 30.12, making it a relatively stable protein, and the fatty acid coefficient is 108.01. The NCBI registration number is AKR15143.1.
2. The MaSglt1 protein had the highest homology with Cypriniformes fish by BLASTP analysis. 54 specific SGLT genes were selected for subsequent phylogenetic tree analysis. In addition, in order to further analyze the characteristics of SGLT protein sequence, we analyzed the conserved motif of SGLT using NCBI, and identified 10 conserved motif from the complete amino acid sequence using MEME. The results showed that most SGLT proteins in the same subfamily have similar motifs, and these observations further validate the close evolutionary relationship of SGLT proteins (Fig. 2). In addition, the sequence similarity of SGLT1 in *Anabarilius grahami* (ROI48931.1), *Ctenopharyngodon idella* (QCF45574.1), and *Sinocyclocheilus rhinocerous* (XP_016374245.1) was 97%, 97%, and 95%, respectively. MaSglt1 protein clustered with SGLT1 of Cypriniformes fish were relatively conserved by phylogenetic tree analysis.
3. Sglt1 protein in aquatic animals contains multiple functional sites that regulate its intracellular transport. SGLT1 sequence of vertebrates is more conserved and is significantly different from the SGLT1 protein sequence of *Escherichia coli* (Fig. 3).
4. Bioinformatics analysis of amino acids composing MaSglt1 protein showed that the protein is a 14-fold transmembrane monosaccharide transporter, with the N-terminal portion located outside the cell and a C-terminal portion located inside the cytoplasm. In terms of its predicted tertiary structure, MaSGLT1 is a transmembrane protein composed of 14 α-helix structures. A hollow channel forms within the protein, which could be specifically coupled with two Na^+^ ions to recognize glucose and carry out transmembrane transport (Fig. 4).

### Tissue distribution of SGLT1

**Figure 5:**
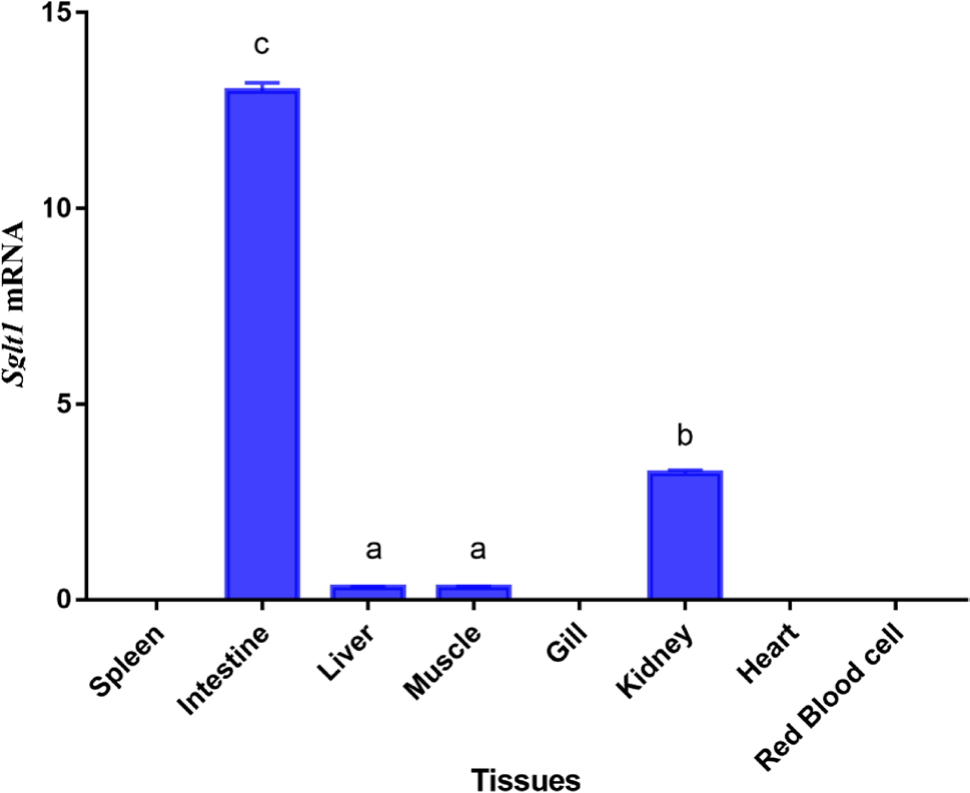
Tissue-specific mRNA expressions of *sglt1* in *Megalobrama amblycephala*. Vertical bars represent mean ± SE values for triplicate samples.

**Figure 6:**
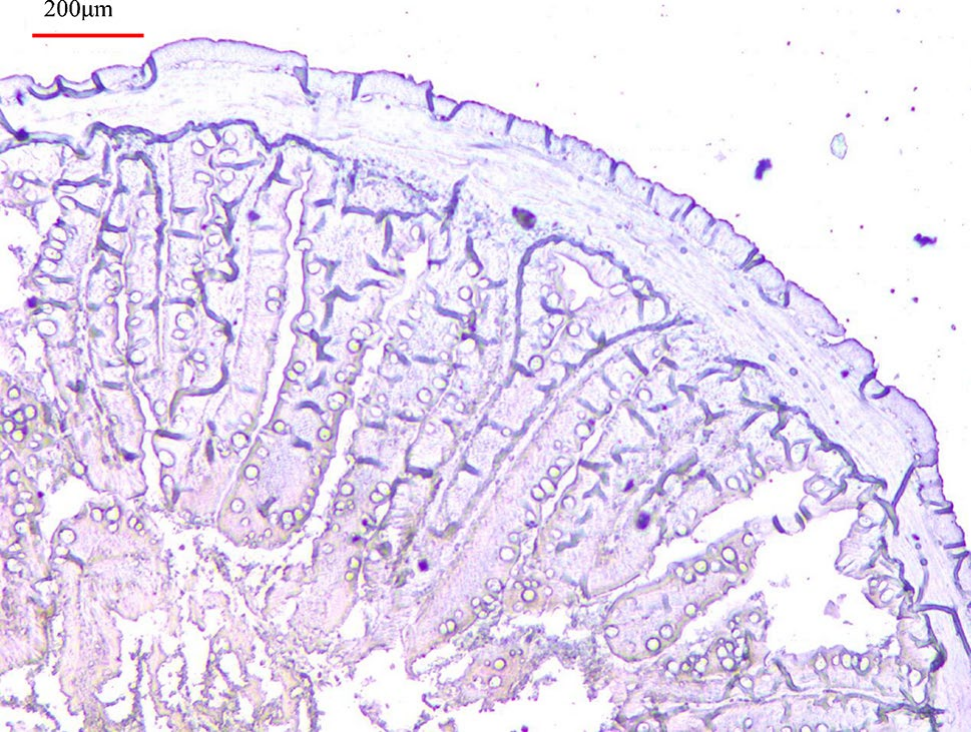
Immunohistochemical staining analysis in intestine of *Megalobrama amblycephala.* The purple signal is our target signal.

High *sglt1* mRNA was observed in intestine and kidney, and highest mRNA level was found in in the intestine. The mRNA in other tissues or organs (liver, muscle, spleen, gill, or heart), as well as in red blood cells, was low or even not detected (Fig. 5). Based on the subcellular localization analysis, *Masglt1* is mainly located in the plasma membrane, accounting for 69.6% of the total expression. In addition, immunohistochemically staining showed that MaSglt1 protein, transporting glucose, is a kind of a membrane protein (Fig. 6).

### Relative expressions of SGLT1

**Figure 7:**
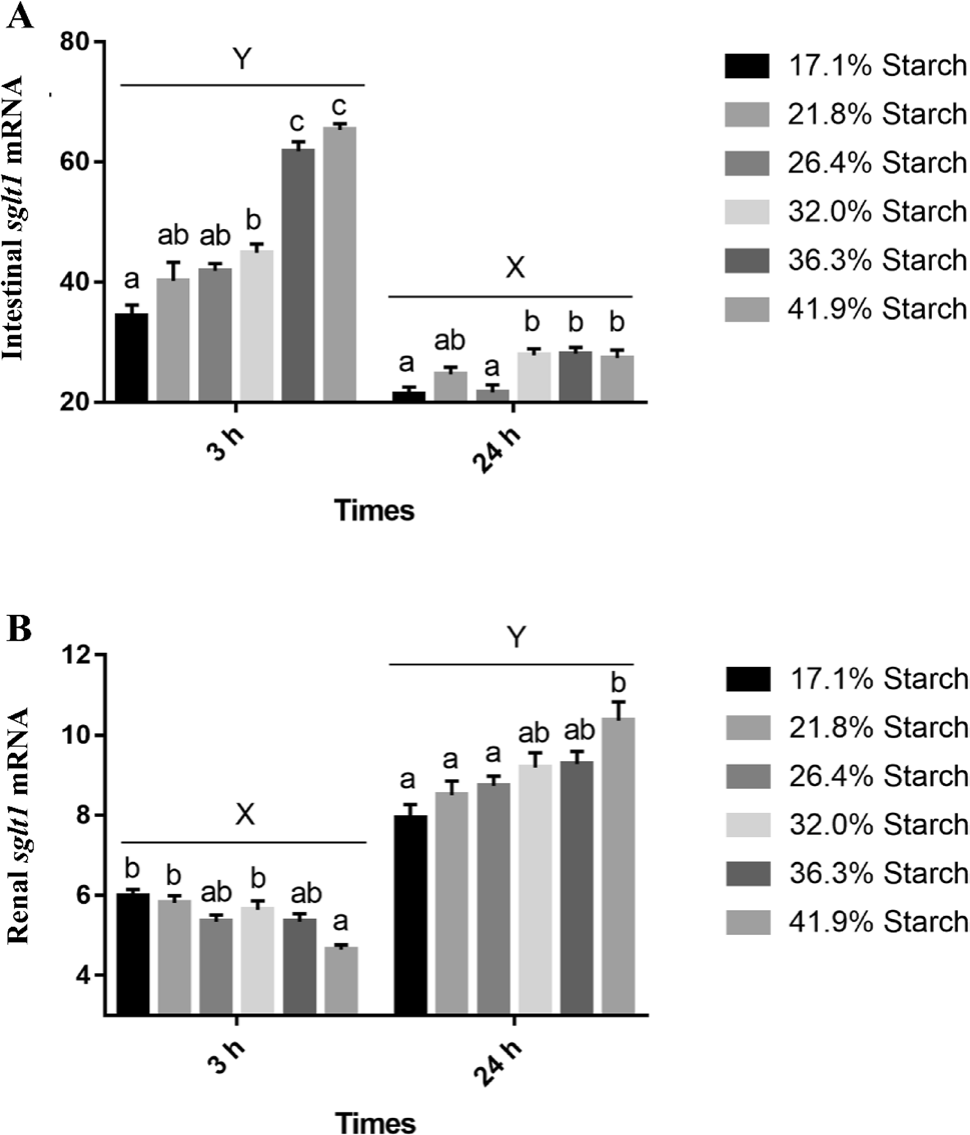
Relative expression of *sglt1* in intestine and kidney in response to different starch levels at 3 h and 24 h after feeding. Vertical bars represent mean ± SE values. Value with the lowercase of different superscripts are significantly different, X and Y represent significant differences at 3 h and 24 h (*P* < 0.05).

The mRNA level of intestinal Masglt1 was increased with the increase of dietary starch levels at 3 h after feeding, and the expression was significantly higher than that at 24 h; however, the mRNA level of renal SGLT1 was opposite that of intestinal *sglt1*. The relative expression of renal sglt1 was increased with the increase of dietary starch levels at 24 h after feeding, and the expression was significantly higher than that at 3 h (Fig. 7).

## Discussion

In this study, this is the first time that *sglt1* cDNA was cloned and identified in blunt snout bream. The full-length cDNA of *sglt1* of *M. amblycephala* (*Masglt1*) (GenBank accession No. KM977634.1) was identified. MaSglt1 protein has high sequence similarity with proteins in other fish species, such as *Anabarilius grahami* (ROI48931.1, 97%), *Ctenopharyngodon idella* (QCF45574.1, 97%), and *Sinocyclocheilus rhinocerous* (XP_016374245.1, 95%). These results showed that the amino acid sequence of this gene was most homologous to sequences in Cypriniformes fish. Furthermore, MaSglt1 protein was clustered onto a branch with the Cypriniformes species, and the evolutionary distance of MaSglt1 in other invertebrates was far from that of vertebrates, while it was completely separated from reptiles, birds and mammals. These results were essentially consistent with the evolutionary status of this species and findings that not only indicated characteristics of direct homologous evolution but also embodied the characteristics of collateral homologous evolution. The cDNA was further categorized as that of *Masglt1* through comparison with other known SGLT1s. Based on the protein 3D structure predictions, MaSglt1 protein is a 14 α-helical transmembrane protein and has specific Na^+^-binding sites. SGLT1 was originally proposed to form 11 transmembrane domains^17^. Subsequent the transport models have deleted or added transmembrane domains^18,20^. In aquatic animals, Sglt1 protein has been shown to be 14 α-helical transmembrane protein in grass carp^28^ and common carp^29^. In addition, SGLT1 protein has also been shown to be 14 α-helical transmembrane protein in human^30,31^ and other animals^32^. These results supported our result. However, there are some reports that proposed models of SGLT1 protein showed 12 membrane spanning domains in rabbit^20^, which may be some differences in the transmembrane domains due to different species. A lot of research of the mechanism on SGLT1 protein has been done by researchers. The three-dimensional conformation of SGLT1 protein indicates that the bottom of the protein can form a hollow channel that can be specifically coupled with two Na ions to recognize monosaccharides and carry out joint transmembrane transport. The empty transporter can bind two sodium ions inducing conformational changes that can improve the affinity of the transporter for the solute; furthermore, another conformational change can also be induced by formation of a ternary complex, which can expose the sodium and substrate to the other site of the membrane. When the substrate and sodium are released from transporter, the empty transporter will reorient and restart the cycle. The sequence of events triggered by sodium release ensures that the ions and substrates are released at the right time^5,20,33–37^. Upon activation, these molecular changes weaken the substrate binding to the transporter, allowing hexose to easily enter the intracellular space^33^ which indicates that glucose may be bound in the center of the core^29^.

Expression analysis of the *sglt1* gene demonstrated its tissue specificity in blunt snout bream which are related to the function and metabolism of tissues^38^. In the present study, the highest *sglt1* mRNA level was observed in the intestine. In mammals, it has also indicated that *SGLT1* gene is mainly distributed in the intestine and is involved in monosaccharide transport^39^. *Sglt1* gene is also mainly distributed in the intestine in aquatic animals such as grass carp^28^, common carp^23^, rainbow trout (*Oncorhynchus mykiss*)^40,41^, gilthead sea bream^22^, North Pacific spiny dogfish (*Squalus suckleyi*)^42^. Based on the results of subcellular localization in and immunohistochemically staining analysis of the intestine, Sglt1 protein was mainly distributed in the intestinal membrane. Numerous studies have confirmed that SGLT1 protein is involved in the accumulation of glucose in intestinal epithelial cells in response to a concentration gradient^15,16^. Since Na^+^ electrochemical potential gradient across the plasma membrane can provide the driving force of Na^+^-coupled transporters, and the route through these transporters also serves as the main uptake pathway of glucose^29^. *SGLT1* gene was originally found in the intestine of rabbits, but it has also been detected in the kidney^43,44^. In this study, *Sglt1* mRNA was also detected in the kidney. Like this study, some studies in mammals and humans have also proven that *SGLT1* is expressed in the kidney, as has been shown in rabbits^44–46^. Some studies have reported that *sglt1* was also expressed in the kidney in aquatic animals such as common carp^23^, shark^24^, skate^25^ and grass carp^28^. SGLT2 protein reabsorbs most of glucose from the glomerular filtrate of kidneys, and SGLT1 protein can recover the rest glucose, preventing glucose loss through urine^1,26^. This may also be the reason for the low expression of SGLT1 in the kidney. Moreover, SGLT1 protein was recently identified as an important glucose transporter of heart and major SGLT isoform was expressed in the heart of mice^47^. Surprisingly, *SGLT1* gene have marginal role in glucose uptake of heart compared to its ability to facilitate glucose uptake^47^, which indicated that, although *SGLT1* gene is distributed in the heart, it may not have played its function properly and may even be nonfunctional in glycometabolism. However, in humans, *SGLT1* gene has an important role in therapeutic target for cardiomyopathy and heart failure^26,48^. Perturbed *SGLT1* expression was observed in diabetic cardiomyopathy and ischemic heart disease, and upregulation of *SGLT1* plays a certain function in improvement in failing left ventricles^49^. This may be due to differences in species. Therefore, the regulatory mechanism of *SGLT1* needs further study.

*SGLT1* gene has been shown to play an important role in the regulation of glucose metabolism^50^. In the present study, the mRNA level of intestinal *sglt1* was increased with the increase of dietary starch levels at 3 h after feeding compared to 24 h after feeding. Furthermore, in our previous study, the glucose levels in the plasma had a positive correlation with dietary starch levels at 3 h after feeding^51^. These results indicated that *sglt1* gene also played an important role in glucose absorption of blunt snout bream through regulating its gene expression in the intestine. Like in our findings, in common carp, the mRNA and protein levels of intestinal Sglt1 increased with increasing dietary carbohydrate levels^52^. In rainbow trout, the mRNA levels of *sglt1* also were increased in response to oral administration of glucose^42,53^. Furthermore, glucose transport was enhanced by carbohydrates in black bullhead (*Ictalurus melas*)^54^. In other animal species, there is increased expression of intestinal *SGLT1* and the capability to absorb monosaccharides in response to increased dietary carbohydrate levels^50,55–58^. In addition, according to some studies, SGLT1 protein is involved in the first step in intestinal glucose absorption^52^ and can cotransport glucose together with sodium through the brush border membrane of enterocytes^59^. The sodium electrochemical potential gradient supplies the energy to drive glucose accumulation in the cell against its concentration gradient^60^, whereas GLUT2 transports glucose from the cytosol to the blood^61^. The expression *GLUT2* tended to be similar to that of *SGLT1* expression^51^, which supports our present results. *Sglt1* gene not only plays a role in intestinal absorption of glucose but also plays a role in renal glucose reabsorption^62,63^. In the present study, the results concerning the mRNA level of renal *sglt1* were opposite to those of intestinal *sglt1*, and the mRNA level of renal *sglt1*1 was increased with the increase of dietary starch levels at 24 h after feeding compared to 3 h after feeding. In our previous study, plasma glucose levels increased after 3 h of feeding dietary carbohydrate, while, plasma glucose levels returning to basal levels after 24 h of feeding of dietary carbohydrate ^51^. Based on results of this study, the reason of increased glucose levels after 3 h of feeding dietary carbohydrate maybe due to increasing intestinal *sglt1* expression to increase glucose absorption, however, when hypoglycemia occurred, the expression of renal *sglt1* was increased to enhance renal glucose reabsorption to prevent glucose loss and maintain blood glucose levels^1^. These results indicated that *sglt1* plays an important role in glucose homeostasis in fish^64^.

In general, the *Masglt1* gene was relatively conserved and was most homologous with sequences in Cypriniformes fish. *Sglt1*gene was mainly distributed in the intestine and kidney and Sglt1 *protein* was located in the cell membrane, where it played a role in intestinal glucose absorption and renal glucose reabsorption.

## Methods

### Ethics declarations

All experiments described above were conducted in compliance with the approved guidelines and regulations of the Institutional Animal Care and Ethics Committee of Nanjing Agricultural University, Nanjing, China [Permit number: SYXK (Su) 2011-0036]. All procedures were approved by the same committee. Study design and was carried out following the ARRIVE guidelines.

### Experimental diets and experimental procedure

The experimental diets and composition referred to our previous study^51^. Furthermore, the diets contained a gradient of starch levels (17.1 (control), 21.8, 26.4, 32.0, 36.3 and 41.9% of dry basis). The experimental procedure was also based on our previous study^51^. In brief, the experimental animals was pre-adult blunt snout bream fish (161 ± 2.5 g), which were cultured in a circulating aquaculture system (1000 L) in triplicate, with each system containing 20 fish. The fish was fed until apparent satiation at 8:00, 12:00 and 16:00, respectively, based on visual observations of feeding behavior. The breeding water temperature was 28.2 ± 0.22 °C, the pH was 7.4 ± 0.09, the dissolved oxygen was 6.9 ± 0.11 × 10^–3^ mg/L, the ammonia nitrogen was 6.9 ± 0.30 × 10^–3^ mg/L, and the hydrogen sulfide was 7.9 ± 0.30 × 10^–3^ mg/L. The photoperiod was maintained according to the standard of 12L: 12D.

### Sample collection

Before breeding, we collected liver, spleen, muscle, intestine, kidney, heart, gill and red blood cell samples from blunt snout bream. These samples were then subjected to expression analysis. After 9 weeks of breeding, samples were collected based on the methods in a previous study^51^. At 3 h and 24 h after feeding, the sampled fish (three fish per tank) were dissected to collect their intestine and kidney based on the results of the tissue expression analysis; the harvested samples were subsequently used for glycometabolism analysis.

### Cloning of SGLT1 cDNA

The cloning method of SGLT1 cDNA was based on our previous study^51^. In a nutshell, (1) extracting the total RNA from a tissue mixtures; (2) synthesizing first-strand cDNA; (3) performing 3′-rapid amplification of cDNA ends (RACE) and 5′-RACE; (4) purifying the PCR products; (4) inserting into a PMD-18 T vector and analyzing sequence. Table 1 shows all the primers used for cloning.

**Table 1:** Sequences of the PCR primers used in this work.

Use	Primer	Primer sequence (5′-3′)
CDS amplification	*sglt1*-F	AGCATCAAAATGGGGGCAGAT
	*sglt1*-R	GATACTGATGTGATCTTTAACC
3′RACE cloning	T3-1	ATGGAAACAGATGAGGTGCGCGAGGAA
	T3-2	TGGCTTTGATCAAGGCAATGCCCCGAA
5′RACE cloning	T5-1	GGTGAAGACAGCAGCA
	T5-2	TAGCTGGTGATGGACTGAATG
	T5-3	CGATACTCACGCCGATCAGAA
Real-time primer	*sglt1*-F	GATTCTCTTATCTCCGCAATG
	*sglt1*-R	GCCACCACACCATACTTC
	*β-actin*-F	TCGTCCACCGCAAATGCTTCTA
	*β-actin*-R	CCGTCACCTTCACCGTTCCAGT

*sglt1*, sodium-dependent glucose cotransporter 1.

### Immunohistochemical analysis

The used antibody of MaSglt1 of immunohistochemically was synthesized based on the cloned *sglt1* gene sequence by Bio-Transduction Lab Co.Ltd. (Wuhan, China). Immunohistochemical analysis was according to our previous study^65^. In brief, the intestinal tissue was encased in resin through paraformaldehyde fixation, alcohol dehydration, and paraffin embedding. Then, a Leica slicer (Lecia, Germany) was used to obtain normal paraffin sections. Finally, immunohistochemically staining was performed through the following process: dewaxing and rehydration, antigen repair, primary antibody incubation, secondary antibody detection, alkaline phosphatase-catalyzed color development, and other processes.

### Quantitative real-time PCR (qRT-PCR) analysis of SGLT1 expression

SGLT1 expression was analyzed as described previously by us^51^. In simple terms, (1) extracting total RNA of tissues; (2) checking the quality and quantity of RNA; (3) operating analysis of SGLT1 expression. The designed primers used for the target gene are shown in Table 1. β-actin was selected for a reference because of relatively stable, which showed that no obvious change of β-actin gene expression was found in the samples of this fish^66^. Pfaffl's mathematical model was used to analyze the relative gene expression for CT calculations^67^.

### Nucleotide sequences and bioinformatics and statistical analyses

Nucleotide sequences and bioinformatics analysis of gene sequences using software or online tools. Details are as follows: (1) ProtParam tool (the basic properties of the proteins), http://web.expasy.org/protparam; (2) PSORT prediction software (subcellular localization analysis), http://psort1.hgc.jp/form.html; (3) NCBI conserved domains (identifying the domain and functionality)^68^, https://www.ncbi.nlm.nih.gov/Structure/cdd/docs/cdd_search.html; (4) On-line analysis (SUMO, phosphorylation and glycosylation), http://www.abcepta.com/sumoplot, http://www.cbs.dtu.dk/services/NetPhos/ and http://www.cbs.dtu.dk/services/NetNGlyc, respectively; (5) ClustalX software (homologous sequence alignment); (6) SWISS-MODEL (predicting the tertiary structure)^69^, https://swissmodel.expasy.org; (7) BLASTP program (similar sequences of amino acid)^70^, NCBI online database; (8) MEGA 7 software (phylogenetic tree)^71,72^; (9) Poisson correction method (calculating evolutionary distance).

The data was subjected to a homogeneity test where necessary. SPSS 16.0 was used to perform statistical analysis of the data with Tukey’s test. The data are expressed as the means with SEMs (M ± SEMs).

## Data Availability

The authors confirm that the data supporting the findings of this study are available within the manuscript, figure and Table.
